# Lung cancer organoids: models for preclinical research and precision medicine

**DOI:** 10.3389/fonc.2023.1293441

**Published:** 2023-10-24

**Authors:** Yajing Liu, Yanbing Zhou, Pu Chen

**Affiliations:** ^1^ School of Pharmacy, Qingdao University, Qingdao, China; ^2^ Research and Development Department, NanoPeptide (Qingdao) Biotechnology Ltd., Qingdao, China; ^3^ Department of Gastrointestinal Surgery, The Affiliated Hospital of Qingdao University, Qingdao, Shandong, China; ^4^ Department of Chemical Engineering and Waterloo Institute for Nanotechnology, University of Waterloo, Waterloo, ON, Canada

**Keywords:** lung cancer organoids, biobanks, drug screening, precision medicine, biomarker exploration

## Abstract

Lung cancer is a malignancy with high incidence and mortality rates globally, and it has a 5-year survival rate of only 10%–20%. The significant heterogeneity in clinical presentation, histological features, multi-omics findings, and drug sensitivity among different lung cancer patients necessitate the development of personalized treatment strategies. The current precision medicine for lung cancer, primarily based on pathological and genomic multi-omics testing, fails to meet the needs of patients with clinically refractory lung cancer. Lung cancer organoids (LCOs) are derived from tumor cells within tumor tissues and are generated through three-dimensional tissue culture, enabling them to faithfully recapitulate *in vivo* tumor characteristics and heterogeneity. The establishment of a series of LCOs biobanks offers promising platforms for efficient screening and identification of novel targets for anti-tumor drug discovery. Moreover, LCOs provide supplementary decision-making factors to enhance the current precision medicine for lung cancer, thereby addressing the limitations associated with pathology-guided approaches in managing refractory lung cancer. This article presents a comprehensive review on the construction methods and potential applications of LCOs in both preclinical and clinical research. It highlights the significance of LCOs in biomarker exploration, drug resistance investigation, target identification, clinical precision drug screening, as well as microfluidic technology-based high-throughput drug screening strategies. Additionally, it discusses the current limitations and future prospects of this field.

## 1 Introduction

Lung cancer is the foremost malignancy in terms of morbidity and mortality worldwide, with a 5-year survival rate of only 10%–20% (1, 2). The disease is characterized by a high degree of heterogeneity in its histology, genomic landscape, and response to therapeutic interventions. According to histopathological classification, lung cancer is primarily categorized into non-small cell lung cancer (NSCLC) and small cell lung cancer (SCLC) (3, 4). Among all lung cancers, NSCLC accounts for approximately 85-88%, while SCLC accounts for the remaining 12-15%. Morever, there exists a diversity of gene mutations among different patients with lung cancer, and the key driver genes of lung cancer also exhibit significant variation (4). The National Comprehensive Cancer Network guidelines recommend testing for a panel of key driver genes in NSCLC, including *EGFR*, *ALK* and *ROS1* (5). Furthermore, the genetic mutations of *KRAS* and *TP53* are pivotal in the pathogenesis of NSCLC. Due to the heterogeneity of tumors, there exist significant variations in patients’ response to specific chemotherapy. Nevertheless, the selection of chemotherapy regimens for lung cancer patients is still largely based on clinical experience, resulting in suboptimal treatment outcomes (6, 7). Currently, an increasing number of targeted drugs are available for lung cancer (8–10). However, the efficacy of these therapies is limited by the absence of reliable biomarkers to predict drug response and the secondary resistance during treatment (11). The current precision medicine strategy, which is based on pathology, gene, and other multi-omics detection results, falls short in effectively addressing the challenging issue of lung cancer. Therefore, it is imperative to explore novel technical approaches or research models to enhance the existing precision medicine strategy.

Conventional approaches to lung cancer research entail the utilization of immortalized lung cancer cell lines, which possess appropriate tumor characteristics can be cultured on a large scale and are amenable to sophisticated analytical techniques, thereby making a significant contribution to cancer research. However, immortalized cell lines may accumulate genetic alterations during prolonged culture, rendering them an inaccurate representation of the original tumor’s genetic properties (12). Monolayer cultured cells display a growth phenotype far removed from that observed in tumors and have limited physiological relevance to human tumors (13). The patient-derived tumor xenografts (PDXs) involve the transplantation of small fragments of surgically excised tumors from cancer patients into highly immunodeficient mice (14). The PDXs often maintain the cellular and histopathological architecture of the original tumor, exhibiting a genomic and gene-expression profile that is remarkably similar to that of the parent patient’s tumor (15–18). However, the application of PDX models is constrained by low success rates, time-consuming procedures, high costs, ethical concerns and species-specific (14, 19). A pressing need exists for an *in vitro* model that accurately preserves the biological characteristics of the original tumor, thereby augmenting the efficacy of lung cancer treatment. The focus of personalized medicine research has shifted towards emerging technologies, such as tumor organoids - three-dimensional structural models composed of multiple cell types *in vitro* that can simulate the structure and function of tumors within a patient’s body (20, 21). The generation of organoids represents one of the most cutting-edge advancements in model development, and they have been successfully derived from a diverse range of tumors (22–25). Lung cancer organoids (LCOs) are capable of faithfully recapitulating *in vivo* tumor characteristics and heterogeneity (26, 27). Furthermore, the response of LCOs to therapeutic drugs is closely correlated with the clinical data of the patients involved, thereby providing supplementary decision-making factors to enhance the current precision medicine system for lung cancer. Compared to PDXs, organoids exhibit a higher success rate in culture, long-term expansion and passage, lower cost and shorter time consumption, making them more suitable for high-throughput drug screening platforms and gene editing technology (28–30). However, LCOs still face several challenges, including low purity (31, 32), lack of specific tumor microenvironment and vascular system (28), as well as issues with standardization and reproducibility of culture, which hinder its broader adoption and implementation in preclinical and clinical research.

In this review, we provide a comprehensive overview of the general approach employed in the construction of LCOs and their potential applications in both preclinical and clinical research. This article will commence with the construction of LCOs, and concentrate on the crucial aspects, current challenges, and potential solutions in the process of constructing LCOs. It elucidates its significance in biomarker exploration, drug resistance research, target identification and drug screening, as well as high-throughput drug screening strategy based on microfluidic technology. Finally, we will address the limitations and future prospects of current organoid applications in lung cancer.

## 2 Methodology for construction of LCOs

With the advancement of 3D culture technology, numerous laboratories have established biobanks of LCOs (33–35). Ebisudani et al. collected lung cancer samples from various sources, including tumor tissue, sputum, and circulating tumor cells, and established a biobank of 43 patient-derived LCOs that accurately recapitulated the histological and molecular characteristics of the original tumors (33). However, the culture success rate of the 37 samples derived from circulating tumor cells was only 8% (3/37). Similarly, only five out of the 25 sputum samples were successfully cultured (5/25). The presence of a limited number of tumor cells in these samples leads to a low success rate in constructing LCOs. In a study conducted by Kim et al., they successfully established 83 non-small cell LCOs with a success rate of 83.0% (83/100), including specimens from malignant pleural effusion, brain metastasis, bone metastasis and primary tumors (34). Wang et al. established a biobank of 160 LCOs mainly derived from malignant serous effusions obtained from 107 patients with various subtypes of lung cancer, including adenocarcinoma, squamous cell carcinoma, small cell lung cancer, adenosquamous carcinoma, sarcomatoid carcinoma (35). The overall success rate for constructing these organoids was 75.7%. And they proposed that lung adenocarcinoma (LUAD) and samples from malignant serous cavity fluid were more favorable for successful culture. The investigation of the relationship between organoid construction and pathology necessitates further studies with larger samples sizes. Data from different research groups often indicated no significant association between organoid construction success rate and pathology. Shi et al. reported success rates of 84.2% (n=16) for LUAD tissue and 93.3% (n=14) for lung squamous cell carcinoma (LUSC) tissue (22). Hu et al. Reported success rates of 77.5% (n=55), 78.3% (n=18) and 100% (n=4) for LUAD tissue, LUSC tissue, and SCLC tissue, respectively (36). Additionally, Wang et al., proposed that insufficient cells was one of the most common factors (35). Similarly, Sachs et al. showed a significant difference in the success rate of organoid culture between surgically resected tissues and biopsy samples. Biopsy samples-derived NSCLC organoids had a modest success rate of only 28% (5/18), whereas surgically resected tissues-derived NSCLC organoids exhibited a high success rate of up to 88% (14/16) (37). Nevertheless, it should be noted that organoids obtained from biopsy samples tend to be more pure compared to those derived from surgical tissues. The methods used to construct the LCOs will be described in detail below.

Successful organoid construction requires three key elements: appropriate cell origin to ensure initial cell activity and sufficient cell numbers, scaffolds to support the 3D spatial structure of the organoids, and culture media that promote both proliferation and differentiation of the organoids. Patient-derived organoids represent the most crucial category of LCOs. These are generated from tumor tissues or cells obtained from the patient’s body, including surgically resected tumor tissues, biopsy specimens, circulating tumor cells, malignant effusions and sputum-derived tumor cells (33). The success rate of LCOs is contingent upon the evaluation of several factors of specimens, including the number of tumor cells in the initial sample, cell viability, degree of tissue necrosis, and pathological type of tumor tissue. The application of scaffolds is a crucial component in facilitating the growth of organoids in 3D mode. LCOs were primarily embedded in Matrigel, which is a material rich in extracellular matrix proteins secreted by Engelbreth-Holm-Swarm mouse sarcoma cells (38). Matrigel not only supports the spatial structure of organoids but also facilitates their formation and differentiation. Although Matrigel currently used in organoid culture is versatile and affordable, its composition is extremely complex with over 1800 unique proteins identified by proteomics analysis alone (39, 40). This complexity can pose challenges in identifying signals necessary for proper organoid structure and function. The variability of Matrigel between batches is significant. The potential immunogenicity of Matrigel, which is derived from mouse cells, has hindered certain studies on human tumor organoids in immunology (41). Currently, synthetic hydrogels are receiving increased attention due to their mechanical properties, functionality and controllable erosion rate. Replacements of Matrigel with synthetic hydrogels are increasingly gaining popularity for the culture of organoids (42, 43). The culture medium serves as a crucial determinant for the successful cultivation of organoids. In terms of lung cancer, the commonly utilized organoid culture medium is composed of two main components: the basal medium and the additive factors. The basal medium consisted of Advanced DMEM/F12 supplemented with HEPES, B27, N2, antibiotics for microbial contamination control, L-glutamine as a nitrogen source, N-acetylcysteine acting as antioxidants and free radical scavengers, and nicotinamide involved in cellular metabolism capacity. The additive factors, including growth factors, pathway inhibitors, and activators, are comprehensively summarized in **Table 1**. Different medium formulations need to be selected for different culture purposes. A study have indicated that cancer organoids cultured in different media may exhibit varying sensitivities to the same drug (46). Therefore, it is crucial to consider experimental culture conditions when correlating functional analysis of LCOs with clinical outcomes.

**Table 1:** Overview of various construction methods associated with human LCOs.

Source of Sample	Digestive enzyme	Additive factors	Reference
Surgically early-stage NSCLC tumor specimens	Liberase TM (1X); TrypLE Express	hEGF (50 ng/mL), hFGF-10 (100 ng/mL), hFGF-4 (100 ng/mL), Noggin (100 ng/mL), A 83-01(0.5 μM), Y-27632 (10 μM), CHIR 99021 (250 nM), and SAG (100 nM)	(22)
Surgical specimens; bronchoscopy biopsies; pleural effusions; circulating tumor cells; sputum samples	Liberase TH (1X)	Gastrin I (10 nM), mEGF (50 ng/mL), hIGF-1 (100 ng/mL), hFGF-2 (100 ng/mL), Noggin (100 ng/mL), R-spondin1 (1 mg/mL), Afamin-Wnt-3A serum-free conditioned medium (25%) and A83-01 (500 nM)	(33)
Malignant effusions and metastatic surgical specimens of advanecd lung adenocarcinoma	Collagenase (2 mg/mL)	Conditioned R-spondin1 medium (20%), hFGF7 (25 ng/mL), Noggin (100 ng/mL), hFGF 10 (100 ng/mL), A83-01 (500 nM), and SB202190 (500 nM)	(34)
Malignant serous effusion; surgically resected biopsies of primary or metastatic leisions	DNase (0.001%), collagenase/dispase (1 mg/mL), penicillin (200 U/mL), streptomycin (200 mg/mL) and amphotericin B (0.5 mg/mL)	bFGF (20 ng/mL), hEGF (50 ng/mL), and Y-27632 (50 ng/mL)	(35)
Surgically resected lung specimens	DNase (0.001%), collagenase/dispase (1 mg/mL), penicillin (200 U/mL), streptomycin (200 mg/mL) and amphotericin B (0.5 mg/mL)	bFGF (20 ng/mL), hEGF (50 ng/mL), and Y-27632 (10μM)	(44)
Surgically lung tumor samples	_	Y-27632 (10 μM), hEGF (50 ng/mL), SB202190 (3 μM), A83-01(5 μM), Forskolin (10 μM) and Dexamethasone (3 nM)	(36)
Surgically NSCLC tumor specimens	_	hEGF (50 ng/mL), Noggin (100 ng/mL), R-spondin 1 (500 ng/mL), FGF-10 (10 ng/mL), FGF-basic (10 ng/mL), Prostaglandin E2 (1μM), Y-27632 (10 μM), A83-01 (0.5 μM), SB202190 (5 μM) and HGF (20 ng/mL)	(45)

As *in vitro* stand-ins for patients, patient-derived LCOs need to maintain important properties of patient tumor tissue, including molecular subtype, histological and phenotypic consistency (22, 33–35, 44). Multiple validations have demonstrated that LCOs can accurately replicate the histological subtypes of lung tumor tissues *in vivo*. Ebisudani et al. have established biobanks encompassing subtypes of lung cancer, including adenocarcinoma, squamous cell carcinoma, small cell lung cancer and large cell neuroendocrine carcinoma organoids (33). Moreover, long-term cultured non-small cell LCOs are capable of maintaining the histological characteristics of their parental tumors. For instance, lung adenocarcinoma organoids can preserve a diverse range of histological subtypes including acinar, lepidic, solid, papillary and mixed types (22). LCOs exhibit genetic mutations, copy number alterations, and aneuploidy patterns that are comparable to those observed in clinical specimens, while largely maintaining the key molecular properties of their parental tumors (33). In tumor tissue, there exist not only aberrantly proliferating neoplastic cells but also no-tumor cells, encompassing immune cells, cancer-associated fibroblasts(CAFs), vascular endothelial cells and other non-neoplastic cells that can be targeted for antitumor therapy (47–51). Dijkstra et al. developed a method capable of co-culturing lung cancer organoids with immune cells, enabling the generation of tumor-reactive T cells by co-culturing non-small cell lung cancer organoids with PBMCs (41). The success rates for generating tumor-reactive CD8^+^ T cell populations ranged from 33 to 50%. Activated CD8^+^ T cells exhibited efficient killing of tumor organoids without the normal tissue organoids. This system provides a valuable tool for investigating the mechanisms sensitivity or resistance to immunotherapy and holds promise for producing patient-specific T cell products for adoptive T cell transfer therapy. The lung cancer organoids were co-cultured with peripheral blood monocytes by Takahashi et al., enabling the *in vitro* evaluation of PD-1 targeted monoclonal antibodies nivolumab and pembrolizumab, both being immune checkpoint inhibitors (52). Another approach developed by Neal et al. involves the utilization of an air-liquid interface (ALI) co-culture system for cultivating non-small cell lung cancer organoids (53). This method enables the preservation of endogenous immune and non-immune interstitial components associated with tumor tissue during organoid construction. A human *in vitro* immunotherapy model was established through uniform culture of tumor epithelium and autologous tumor-reactive tumor-infiltrating cells. Tumor-infiltrating lymphocytes of human and mouse tumor organoids demonstrated functional activation, expansion, and cytotoxic responses to PD-1/PD-L1 checkpoint blockade as evaluated through a 7-day rapid assessment. However, a limitation of this technique is that tumor-infiltrating lymphocytes cannot be maintained in the culture medium for more than 60 days. The formation of tumor blood vessels creates a malignant tumor microenvironment within the body, providing nourishment to tumors and promoting both tumor progression and drug resistance (54, 55). Inhibiting angiogenesis is a crucial strategy in the treatment of tumors. Seitlinger et al. presented a methodology for the vascularization of lung cancer organoids, wherein human lung fibroblasts were incorporated into NSCLC patient-derived tumor cells to generate more intricate tumor organoids that mimic spatial organization (56). Subsequently, these tumor organoids were vascularized using primary human endothelial and connected to pre-vascularized fibrin hydrogel, thereby simulating the authentic vascular network within the tumor and its microenvironment. This approach holds promise for integration with microfluidic chips in order to evaluate drug efficacy. Nashimoto et al. present a lung cancer organoid chip integrated with a perfusable vascular network, which, when combined with an electrochemical sensing platform, enables the evaluation of oxygen metabolism changes in LCOs before and after drug administration (57). Furthermore, 3D bioprinting is a crucial technique in the field of vascularization strategy. Choi et al. presented an advanced model for vascularized LCOs, which consists of LCOs, lung fibroblasts, and a perfusable vascular network created through 3D biopanning (58). This model allowed for the evaluation of drug responsiveness in a vascularized LCOs. CAFs play a crucial role in various biological processes of cancer, including cancer initiation, progression, drug resistance, and distant metastasis (47). Sen et al. established an organoid model of SCLC with fibroblasts, which validated the paracrine effects of fibroblasts in promoting faster and stronger regeneration of SCLC cells (59). This model provides a valuable platform for targeted drug screening to identify novel therapeutic strategies for SCLC. A 3D co-culture system incorporating extracellular matrix and CAFs can effectively recapitulate the progression of lung squamous cell carcinoma, providing a valuable tool for investigating the dynamic interplay between tumor cells and stromal components (60). Utilizing this model, it was demonstrated that CAFs are capable of inhibiting *SOX2* function while promoting the proliferation of patient-derived non-small cell lung cancer (NSCLC) cells. 3D co-culture models that incorporate patient-derived organoids and CAFs hold great promise as a means of capturing the heterogeneity and complexity of primary tumors, making them valuable tools for investigating more effective treatment regimens within the tumor microenvironment.

Tumor organoids for clinical precision medicine require the reliable establishment of pure tumor organoids to obtain more accurate drug screening and genetic testing data. Currently, surgically resected lung specimens are the primary source of LCOs, but these specimens contain a diverse range of cells including not only tumor cells but also a significant number of normal lung epithelial and interstitial cells. With the aforementioned culture methods and conditions, airway epithelial cells of normalcy can be derived from lung cancer tissue and subsequently undergo excessive proliferation. Dijkstra et al. identified 70 organoids from NSCLC samples using a genetic testing method, and determined that only 17% of the cultures were pure non-small cell LCOs while 80% showed normal airway overgrowth (32). Furthermore, distinguishing between these two types of organoids is not possible through simple histomorphological methods, rendering manual removal of normal morphology organoids an unsuitable method for purifying LCOs. In order to inhibit the growth of normal airway and alveolar organoids, various media formulations have been developed based on the differential reliance on growth factors between normal and cancerous cells. For instance, since normal airway and alveolar organoids are unable to proliferate in the presence of *ERBB* inhibitor, this condition hinders *ERBB* signaling by eliminating EGF, insulin growth factor-1, fibroblast growth factor-2 while introducing a pan-ERBB inhibitor to prevent formation of normal airway organoids (33). Alternatively, based on the high frequency of *TP53* mutations in lung cancer, the addition of Nutlin-3 (an MDM-2 inhibitor) to the culture medium effectively suppressed normal organoids and enriched *TP53*-mutant LCOs (33, 61). However, this approach also inhibited the formation and growth of *TP53* wild-type LCOs, resulting in a partial loss of heterogeneity. It is noteworthy that Hu et al. have reported a mechanical treatment method, which involves gentle grinding followed by filtration through a 100 µm filter using a syringe and collection of tumor fragments ranging from 40 to 100 µm with the aid of a 40 µm filter (36). Late culture using growth factor-deficient medium for LCOs, which lack essential factors such as FGF7, FGF10, Rspondin-1 and Noggin, resulted in a significant increase of tumor cell proportion from 49 ± 15% in tumor tissue to 78 ± 17% in all no-passaged LCOs. This approach has been demonstrated to facilitate rapid formation of large LCOs within 24 h while minimizing contamination by mixed cells. The purity of organoids is significantly affected by the type of sample. Organoids derived from malignant ascites are preferred due to their mainly tumor cell composition, which results in more purer tumor organoids and makes them excellent candidates for drug sensitivity testing (33–35). Both tissue and malignant ascites-derived LCOs effectively reflect the pathological and molecular characteristics of primary tumors, providing a reliable foundation for subsequent drug sensitivity testing (35). While some progress has been made in purifying LCOs, further exploration is necessary to develop an easy-to-use purification method with higher purity that fully preserves the heterogeneity of the original tumor.

The process of normal cell transformation into tumor cells necessitates a sequence of genetic mutations, including the activation of oncogenes or the inactivation of tumor suppressor genes (62). Naranjo et al. generated LCOs harboring specific mutations by introducing alterations in key genes, such as *KRAS*, *BRAF*, and *ALK*, into AT2 cells-the initiating cell type of mouse lung adenocarcinoma (63). LCOs can also be derived from various pluripotent stem cells, such as human embryonic stem cells, lung epithelial progenitor cells, induced pluripotent stem cells (64–66). The utilization of gene-edited LCOs, presents an opportunity to investigate the initial stages of lung cancer and how genetic damage triggers carcinogenesis (66). These non-tumor cell-derived models offer a valuable research tool for exploring the relationship between gene mutations and cancer initiation, as well as the biological behavior of tumors.

## 3 LCOs for preclinical research

**Figure 1:**
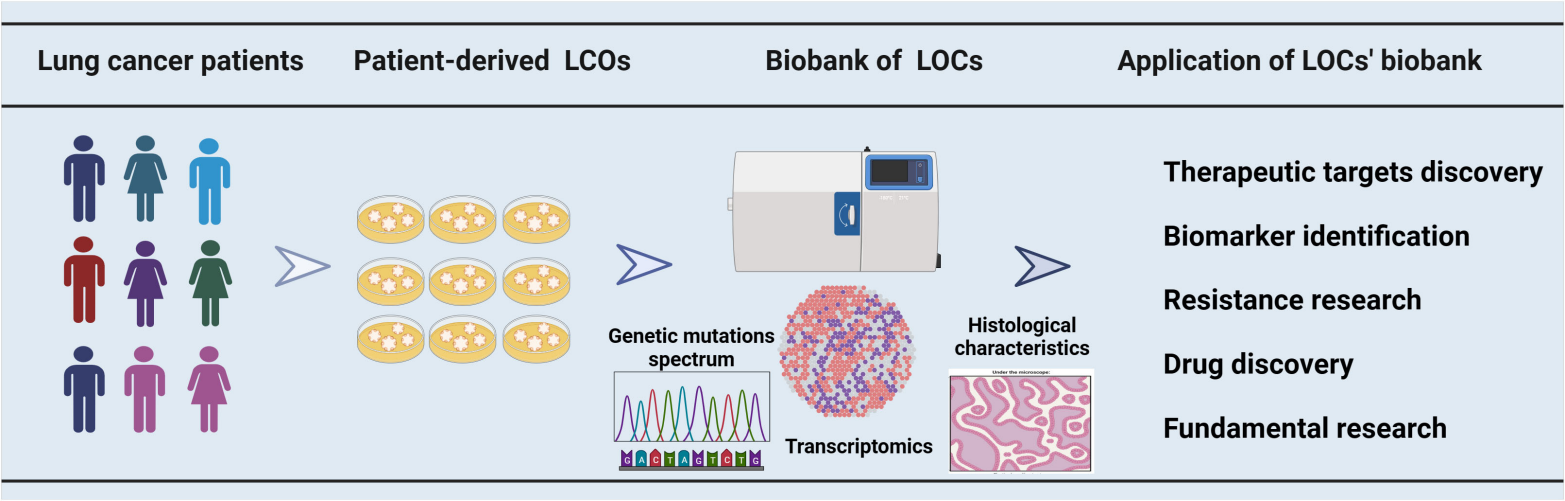
The utilization of a biobank containing organoids derived from lung cancer patients in cancer research. A biobank comprising multiple samples was established using LCOs derived from patients with lung cancer. The LCOs biobanks for lung cancer were characterized through gene sequencing, transcriptome analysis, and histological examination. These biobanks have a broad range of applications, encompassing therapeutic target discovery, biomarker identification, drug resistance research, drug development and some fundamental research.

Lung cancer is a disease characterized by heterogeneity, which results in diverse responses to drugs among patients. Genetic detection methods can assist in selecting targeted therapy for lung cancer patients; however, the complexity of gene mutations and the lack of reliable biomarkers contribute to primary or secondary drug resistance, leading to suboptimal overall patient benefit rates (67). The LCOs represent an *in vitro* culture model that can faithfully recapitulate the characteristics of tumors. Some research groups have established LCO-based biobanks, which provide a valuable tool for identifying biomarkers (33), discovering therapeutic targets (68) and investigating drug resistance (69) (**Figure 1**).

### 3.1 LCOs for identifying biomarkers

Predictive biomarkers commonly evaluated in cancer therapy include specific protein expression levels, somatic DNA alterations in a single gene, genome-wide patterns of somatic DNA alterations, and populations of non-tumor cells that shape the tumor microenvironment (70). Biomarker-matched therapies have demonstrated significant survival benefits in lung cancer patient (71, 72). However, despite the development of biomarkers in cell and animal models, less than 1% of cancer biomarkers published are ultimately translated into clinical practice (71, 73). LCOs have the ability to replicate the genetic characteristics and protein expression of individual patients, making them a promising tool for predicting treatment outcomes through biomarker analysis. The identification of new biomarkers based on the relationship between niche factor-dependent phenotypes and genotypes in LCOs represents an innovative approach. A chemically defined serum-free medium was utilized to investigate the impact of growth factors on LCOs proliferation. Through gene sequencing techniques, Ebisudani et al. discovered that loss of *NKX2-1* resulted in Wnt-3A and R-spondin dependence in lung adenocarcinoma organoids (33). Further investigation revealed that the porcupine inhibitor (C59) targeting Wnt signaling pathway could specifically target *NKX2-1* deficient LCOs and effectively inhibit their proliferation. The Wnt signaling pathway is a promising drug target for lung cancer, and the efficacy of Wnt inhibitors (porcupine inhibitors) is currently being validated in multiple phase I clinical trials. However, drug resistance has been observed in some patients. Therefore, from a clinical perspective, *NKX2-1* expression identified by LCOs has the potential to serve as a practical biomarker for predicting response to Wnt-targeted therapy in lung cancer. LCOs can be combined with genetic data to identify novel biomarkers. The high frequency of *KRAS* mutation in lung cancer is often associated with increased invasiveness, poor prognosis, and drug resistance (74–76). Tyc et al. employed a genetic assay to identify KDS30, a marker comprising 30 gene expression signatures in KRAS mutation-dependent tumors (77). The combination therapy of neratinib (an EGFR/ERBB2 inhibitor) and cobiotinib (a MEK inhibitor) exhibited synergistic anti-tumor proliferation exclusively in organoids derived from patients with high KDS30 mt *KRAS* rather than low ones. Organoids, as *in vitro* models of patients, possess inherent advantages when combined with cutting-edge biotechnology to obtain valuable predictive biomarkers (78). Utilizing CyTOF, a single-cell detection technique, Taverna et al. stratified cells based on the expression levels of *AXK* and *JAK* and employed LCOs to evaluate the efficacy of *AXL* and *JAK* inhibitors (45). LCOs with moderate to high levels of AXL and JAK proteins exhibited heightened sensitivity to TP-0903 (an AXL inhibitor) and ruxolitinib (a JAK inhibitor), whereas those with low expression failed to respond. Analysis of The Cancer Genome Atlas patient samples revealed that chromosome 3p24, which encompasses *RBMS3*, is frequently lost in NSCLC and correlates with poor prognosis. CRISPR/Cas9-mediated *RBMS3* knockdown promotes the growth of *BRAF* ^V600E^-driven lung organoids (79). Silencing of *RBMS3* confers resistance to dabrafenib/trametinib but sensitivity to porcupine inhibition in *BRAF* ^V600E^-mutant lung tumors, suggesting that *RBMS3* silencing and *BRAF* ^V600E^ mutation may serve as predictive biomarkers for drug response. LCOs, as a preclinical tumor model with similar molecular characteristics to tumor samples while minimizing the confounding effects of miscellaneous cells on test results, can be combined with high-throughput RNA sequencing (RNA-Seq) to identify *IRAK1BP1* as a novel disease predictor in lung adenocarcinoma (80). The loss of *IRAK1BP1* is associated with poor prognosis in patients with lung adenocarcinoma. Biomarker-based stratification of patients enables identification of individuals with specific characteristics that would benefit from treatment, thereby enhancing overall therapeutic efficacy. Patient-derived LCOs generated from malignant pleural effusions were utilized to assess drug sensitivity and distinguish the impact of *MET* dysregulation on first-line therapy in *EGFR*-mutated patients (81). Patients were stratified into two groups: *EGFR*+/*MET*amp- (n = 22) and *EGFR*+/*MET*amp+ (n = 18). The clinical outcomes of untreated patients with advanced non-small cell lung cancer in the *EGFR*+/*MET*amp+ group were compared to those in the *EGFR*+/*MET*amp- group. Dual targeted therapy was found to be more effective than tyrosine kinase inhibitor (TKI) monotherapy for patients in the *EGFR*+/*MET*amp + group. In the realm of lung cancer research, a plethora of biomarkers have been identified and deemed efficacious; however, their clinical validation remains elusive (71). *FGFR1* amplification is not a reliable biomarker for *FGFR* inhibitors in treating lung squamous cell carcinoma due to its limited efficacy with only 7% to 11% of patients exhibiting durable responses in clinical trials (82, 83). Shi et al. proposed the utilization of LCOs characterized by *FGFR1* amplification for evaluating the efficacy of combination therapy regimens (22). The co-administration of *FGFR* inhibitor and *MEK* inhibitor (trametinib) exhibited a potent synergistic effect, targeting pFGFR, pAkt, and pErk, thereby effectively inhibiting tumor organoid proliferation. Amplification of *FGFR1* as a biomarker supports the use of combined *FGF*R and *MEK* inhibitors in lung squamous cell carcinoma.

### 3.2 LCOs for discovering targets

The identification of novel therapeutic targets is a crucial foundation for the development of new drugs. However, conventional lung cancer cell lines utilized in research often present challenges in identifying valuable therapeutic targets due to limited cellular diversity, loss of spatial organization and tumor microenvironment, as well as gradual loss of tumor specificity during prolonged culture and alterations in gene expression profiles (84). LCOs posses inherent advantages in the identification of novel therapeutic targets due to their ability to faithfully replicate crucial characteristics of lung tumors. Ma et al. utilized non-small cell LCOs to identify *CDK1*, *CCNB2*, and *CDC25A* as pivotal oncogenes in lung adenocarcinoma but not in lung squamous cell carcinoma (68). Subsequent knockdown experiments targeting *CDK1* and *CCNB2* using both adenocarcinoma cell lines and LCOs demonstrated their inhibitory effects on the proliferation of lung adenocarcinoma. Knockdown of *CDC25A* did not impede the proliferation of lung adenocarcinoma cell lines, but effectively suppressed the growth of lung adenocarcinoma organoids. These findings are likely attributed to differential gene expression between 2D and 3D cultures. *CDK1*, *CCNB2*, and *CDC25* may serve as promising therapeutic targets and potential biomarkers. Fascin, a pro-metastatic actin bundling protein upregulated in all metastatic cancers, promotes tumor growth and metastasis by increasing glycolysis in lung cancer. Lin et al. reported that pharmacological inhibitors of Fascin can effectively inhibit YAP1-PFKFB3 signaling and glycolysis in LCOs, thereby inhibiting tumor growth and metastasis (85). These findings suggest a promising therapeutic target for lung cancer.

### 3.3 LCOs for studying drug resistance

Many cancer patients initially respond well to drug treatment, but eventually develop resistance through complex mechanisms such as drug efflux, DNA damage repair, inhibition of cell death, and DNA mutations (86–89). In order to further advance the development of new generation anti-tumor drugs, an *in vitro* drug-resistant tumor model is crucial for preclinical drug evaluation. Tumor organoids also offer significant advantages in addressing lung cancer drug resistance due to their ability to accurately replicate the epigenetics, genetic profiling, and histopathology of tumors *in vivo*. Banda et al. introduced erlotinib, an *EGFR* inhibitor commonly used in lung cancer treatment, into organoids culture and observed a significant enrichment of at least one known therapy-resistant mutation (*BRAF* ^V600E^, *KRAS* ^G12D^, *KRAS* ^G12V^, and *PIK3CA* ^H1047R^) associated with erlotinib after prolonged culture (69). They established an erlotinib resistance model for lung adenocarcinoma organoids that can be utilized to simulate tumors with various mutation subsets. Organoids can serve as tools for investigating drug resistance mechanisms and developing novel therapeutic strategies. Han et al. utilized patient-derived non-small cell LCOs to explore drug resistance and found that elevated expression of tumor *CD73* in patients with *EGFR* mutation contributes to the immunologically quiescent microenvironment of *EGFR*-mutant NSCLC, leading to immune checkpoint therapy resistance (90). Yan et al. demonstrated the crucial role of *DCLK1* in maintaining tumor cell stemness properties, as well as its high expression in EGFR-TKI-resistant LCOs (91). Furthermore, they found that *DCLK1* inhibitors can reverse this secondary resistance to TKI. Therefore, for lung adenocarcinoma patients with EGFR-TKI (gefitinib, erlotinib) resistance, *DCLK1* inhibitors may serve as a promising alternative treatment option. Combination therapy or multidrug therapy is a promising approach for treating lung cancer and eradicating mutant subpopulations that cause drug resistance. Tumor organoids can provide a more accurate representation of drug resistance occurrence *in vivo* and facilitate the development of new drug combinations that effectively prevent tumor growth and overcome drug resistance. Glutathione S-transferase pi (GSTP1) is a phase II detoxification enzyme that is highly expressed in lung cancer and mediates chemotherapy resistance (92). The combination of ezatiostat, a specific GSTP1 inhibitor, and crizotinib, an ALK inhibitor, can regulate the activity of lung cancer stem cells. This combined treatment has demonstrated significant inhibitory effects on the proliferation of TKI-resistant lung adenocarcinoma organoids. The development of sensitizing agents is a crucial strategy in combating drug resistance. Manoalide, a natural inhibitor of PLA2, has been identified as a potential EGFR-TKI sensitizer for *KRAS*-mutated and osimertinib-resistant lung cancer organoid by inhibiting the KRAS-ERK signaling pathway (93). Cisplatin, a first-line chemotherapeutic agent for lung cancer treatment, often leads to drug resistance in patients (94, 95). Li et al. discovered that halofuginone, a natural compound, can sensitize cisplatin-resistant LCOs by inducing G0/G1 phase arrest and promoting apoptosis through PI3K/AKT and MAPK signaling pathway inhibition (96). This finding may improve the prognosis of cisplatin-resistant lung cancer patients.

## 4 The application of LCOs in drug screening and precision medicine

**Figure 2:**
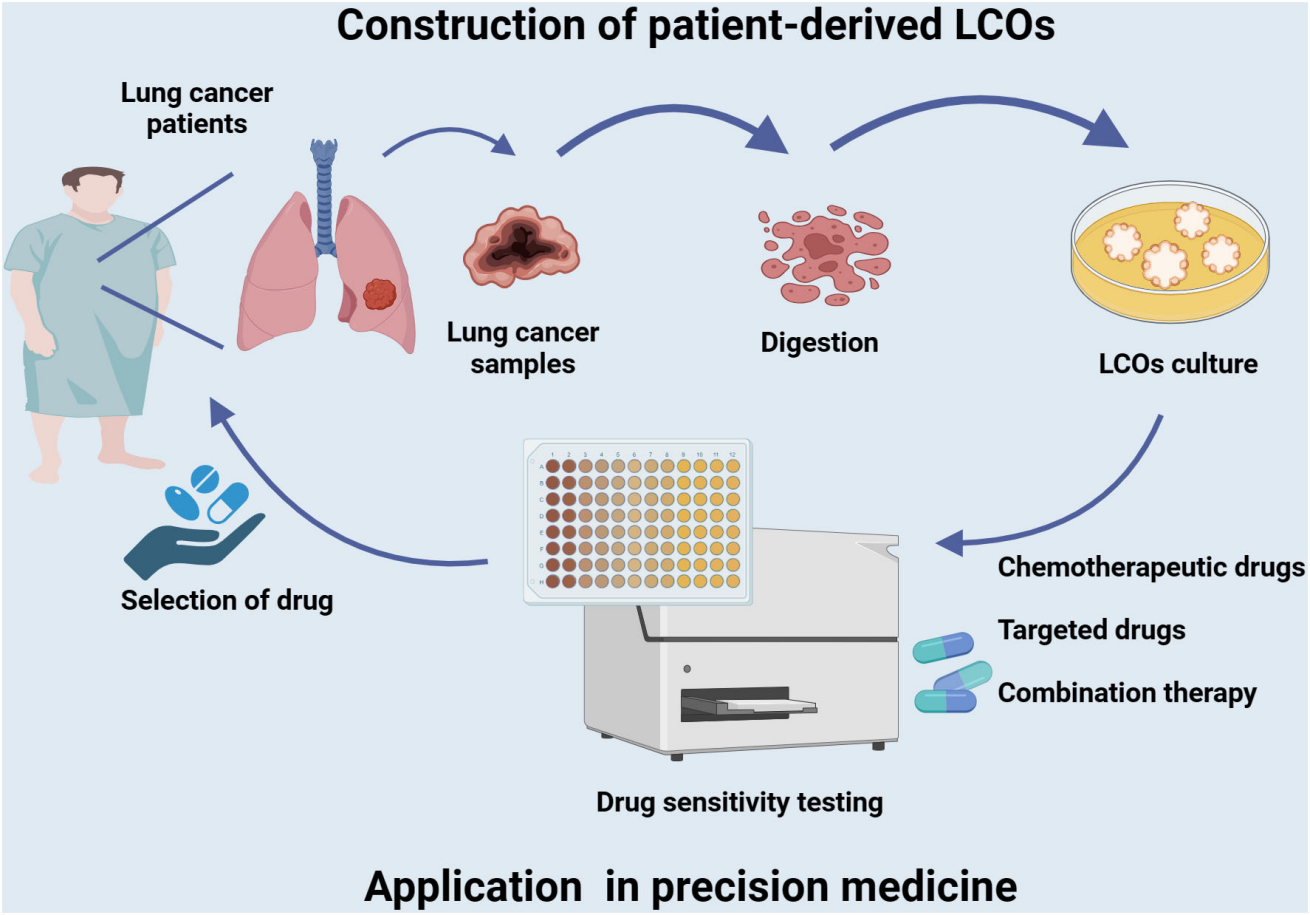
Establishment of LCOs and its application in precision medicine. Tumor tissues of lung cancer patients are collected to generate LCOs. The LCOs can be utilized directly for drug screening of a variety of commonly used first-line and second-line drugs, including chemotherapy agents, targeted therapies, or combination regimens. Corresponding sensitive drugs are selected to develop a personalized treatment plan based on the patient’s characteristics and facilitate precise treatment.

Chemotherapy and radiotherapy are established treatment modalities that serve as the standard of care for a variety of cancers. However, many antitumor therapies are associated with toxicity and non-response (97). Tumor organoids can be utilized to identify the direct impact of antineoplastic drugs on cancer cells, thereby distinguishing effective from ineffective treatments (**Figure 2**). In a real-world study, Wang et al. established a biobank of living LCOs derived from malignant ascites of patients with lung cancer, and demonstrated its efficacy in predicting patient response (35). The sensitivity of organoids to osimertinib, chemotherapy, dual targeted therapy and other targeted therapy was 86.7%(13/15), 83.3%(10/12), 100%(10/10) and 70.6%(12/17), respectively. The overall sensitivity and specificity of the test were 84.0% (95%CI, 63.08%-94.75%) and 82.8% (95%CI, 63.51%-93.47%), respectively, with an accuracy rate of 83.3%. LCOs have the potential to prevent unnecessary treatment for patients who are unlikely to benefit from it. Further prospective clinical trials are required to investigate the feasibility of organoid-guided therapy for lung cancer patients. The subsequent systematic review focuses on the utilization of LCOs as *in vitro* models to assess drug sensitivity, encompassing chemotherapeutic agents and targeted drugs against common lung cancer biomarkers such as *EFGR*, *ALK*, and *KRAS*.

EGFR is a tyrosine kinase receptor that triggers the activation of multiple downstream pathways, including the RAS/MAPK, PI3K/AKT, and JAK/STAT pathways (98–100). It plays a pivotal role in regulating cellular processes such as proliferation, survival, adhesion, migration, and differentiation. Overexpression of *EGFR* and dysregulation of its signaling pathways have been observed in various types of cancer, particularly NSCLC (101, 102). Wang et al. utilized LCOs derived from malignant ascites of patients to predict the clinical response to osimertinib, an *EGFR* mutation-targeting agent (35). Based on both the clinical treatment outcomes and drug sensitivity test results of corresponding patient-derived LCOs, it was observed that the IC_50_ value for progressive disease (PD) group was significantly higher than that for partial response (PR) group. The confirmation of the correlation between tumor volume reduction and IC_50_ value suggests that this approach may serve as a predictive tool for the efficacy of targeted agents in treating *EGFR* mutations-associated lung cancers. There exist eight prevalent *EGFR* mutations, encompassing DeletionExon19, DeletionExon21, G719X mutation, L861Q mutation, L858R mutation, S768I mutation, T790M mutation and Insertion-Exon20 mutation (103). Bie et al. reported that organoids could also serve as a valuable tool for assessing the sensitivity of rare *EGFR* double mutations to *EGFR*-TKIs (104). A case study involved that patient-derived non-small cell lung cancer organoids containing the rare *EGFR* 19Del/L643V double mutation type organoid, which was found to be responsive to osimertinib and gefitinib but resistant to erlotinib and icotinib, highlighting the importance of personalized treatment for patients harboring rare *EGFR* double mutations. Kim et al. showed that two LCOs with same *EGFR* p.L858R mutant exhibited varying degrees of sensitivity to the c-Met inhibitor crizotinib (44). It is noteworthy that certain organoids may still manifest sensitivity to *EGFR*-TKI, despite the absence of *EGFR* mutations (103). In a female Asian NSCLC patient, common *EGFR* mutations were not detected in either primary tumor tissue or biopsy-derived PDO. The genetic test report did not indicate sensitivity to *EGFR* TKIs, such as gefitinib and erlotinib. However, the patient’s PDO drug sensitivity test results revealed that she was sensitive to gefitinib. Following treatment with gefitinib, the patient’s prognosis significantly improved. This underscores the importance of functional drug testing using LCO and studying resistance mechanisms through organoids research in order to better predict clinical response to drugs.

NSCLC is the most prevalent tumor associated with ALK gene fusion. Approximately 5% of NSCLC patients exhibit positive results for ALK fusion, with EML4 being the primary fusion partner (105, 106). The patient was initially diagnosed with EML4-ALK fusion (35). Following resistance to second-generation *ALK*-TKIs, ceritinib and SAF-189s, alectinib was administered as third-line targeted therapy. However, organoid drug susceptibility testing revealed a lack of sensitivity to alectinib, ultimately leading to the development of intracranial metastasis and disease progression. The clinical efficacy of targeted therapy in *ALK* gene fusion lung cancer patients was validated, and the drug susceptibility detection results from all organoids were consistent with the clinical response (100%, 5/5). Despite a small sample size, this study highlights the potential for tumor organoids to serve as *in vitro* surrogates for patients.

*RAS* mutations are frequently observed in gastrointestinal and lung malignancies, with *KRAS* mutations being the most prevalent subtype (107). *KRAS* mutations account for approximately 78% of all *RAS* mutations detected in NSCLC (108, 109). *KRAS* mutant tumor cells have been shown to exhibit greater sensitivity to MEK inhibitors compared to *KRAS* wild-type cell lines (110). This finding is consistent with preclinical studies conducted on *KRAS* mutant LCOs, which demonstrated that LCOs carrying the *KRAS* mutation were more responsive to trametinib, a MEK inhibitor, than their wild-type counterparts. Specifically, the IC_50_ value for *KRAS* mutant organoids was lower than wild-type counterparts (22). The MEK inhibitor selumetinib yielded similar results. Although not yet validated in clinical treatment outcomes, these findings support the use of organoids as a promising tool for preclinical drug screening.

The occurrence of resistance to individual anticancer agents is prevalent among patients with advanced lung cancer (111, 112). Due to the intricate mechanisms underlying drug resistance, it is challenging to satisfy clinical demands through monotherapy. The utilization of LCOs holds significant potential in guiding combination therapy efficacy prediction *in vitro*. Wang et al. reported that the patient’s tumor exhibited molecular characteristics of *EGFR* ^L858R^ mutation and *MET* copy number gain, and despite treatment with osimertinib monotherapy, disease progression persisted (35). In this patient-derived LCOs model, the IC_50_ value for osimertinib in combination with itself was lower than that observed for either agent alone. The patient exhibited a PR to a treatment regimen consisting of osimertinib and vorolitinib. These cases suggest that LCOs have the potential to predict effective combination therapies.

Monoclonal antibody blockade is the most direct targeted therapy for *EGFR* which overexpressed in 85% of NSCLC (102, 113). Cetuximab’s ability to inhibit *EGFR* signaling and lead to growth inhibition is due to the high expression of *EGFR* and *EGF*-dependent growth of LCOs (106). *HER2* can activate the same key signaling pathways as *EGFR*, making it an ideal target for anticancer drugs. The sensitivity of three LCOs to monoclonal antibody drugs targeting tumor cells, including trastuzumab, pertuzumab, and trastuzumab emtansine, was assessed using a 384-well plate high-throughput screening system (52). Trastuzumab did not exhibit any inhibitory effect on the tested organoids; however, trastuzumab emtansine, an antibody-drug conjugate (ADC) targeting microtubules, demonstrated potent cytotoxicity against each LCOs. The degree of inhibition correlated with the level of HER2 protein expression. The LCOs models represent a valuable tool for screening antibody-based therapeutics and the drug sensitivity profiling supports the clinical potential of ADCs as efficacious anti-tumor agents.

The efficacy of chemotherapy drugs commonly used in the treatment of lung cancer is generally limited, and they also carry significant risks of adverse effects (6, 114). Therefore, it is crucial to determine appropriate medication strategies for individual patients and identify those who are most likely to benefit from such treatments. Wang et al. reported on the treatment of lung cancer patients with a combination therapy involving both etoposide and cisplatin, referred to as EP therapy (35). Despite variations in disease subtypes, including lung adenocarcinoma and small cell lung cancer, drug sensitivity testing conducted on LCOs demonstrated consistent sensitivity independent of disease pathology, accurately predicting clinical response to the EP regimen. The *in vitro* detection of LCOs may serve as a reliable indicator for the clinical response of patients to chemotherapy agents.

## 5 LCOs integrated on a microfluidic chip

Due to the limited number of viable tumor cells obtained from patient samples and the use of conventional cell culture techniques operating in microupscaling volumes, drug testing using organoids may take weeks or even months before providing results to patients (115, 116). Moreover, in comparison to the standard cell culture system, tumor organoids suffer from several drawbacks including high cost, low throughput, complex operation and poor repeatability, which ultimately reduces the reliability and accessibility of tumor organoids as a tool for predicting clinical responses. To surmount these technical challenges, an approach to address these issues is to establish an organoid drug susceptibility testing platform utilizing microfluidic technology (117, 118). Jung et al. employed soft lithography technology to produce a cost-effective, one-step 3D microfluidic platform. The device featuring 29 wells was infused with Matrigel and seeded with patient-derived LCOs for drug sensitivity testing of cisplatin and etoposide via flow medium culture (119). Organoids exhibiting faithful recapitulation of tumor characteristics and uniform size distribution were found to yield reliable and accurate drug responses. To meet the patient’s time constraints and shorten testing duration, Hu and colleagues have developed an integrated superhydrophobic microwell array chip (InSMARchip) as a replacement for conventional 96-well culture plates in drug susceptibility testing of LCOs at the nanoliter scale (36). 3-day-old no-passaged organoids were seeded into InSMARchip and subjected to a three-day drug susceptibility test. A set of drug tests recommended by clinical practice guidelines can be completed within a week without requiring prolonged amplification time. Furthermore, analysis of patient samples has demonstrated robust associations between reported drug responses and genetic mutations as well as clinical outcomes. The research group subsequently developed a vitrification-based freezing method for the *in situ* cryopreservation of LCOs (120). The tumor organoids are frozen on smart chips and can be stored in a liquid nitrogen tank for a long time. After thawing, drug sensitivity testing can be resumed with minimal damage to LCOs induced by freezing and thawing. The cryopreserved chip is now ready for subsequent high-throughput drug screening applications, providing convenience in the use of LCOs.

## 6 Current challenges and perspectives

Although LCOs hold great promise as a drug screening tool, potential biomarker library, and model for drug evaluation, this advanced model is not without limitations.

1. Cells within the tumor microenvironment, including CAFs, adipocytes, endothelial cells, and immune cells, play a pivotal role in the initiation and progression of cancer (121–123). These cells associated with cancer regulate various aspects of cancer cell behavior such as proliferation, migration, invasion and apoptosis through direct cell-to-cell interactions as well as secretion of soluble factors, extracellular matrix components and small metabolites (47, 124, 125). Therefore, to better replicate cancer progression and drug response *in vivo*, it is imperative to incorporate these components into an *in vitro* model. However, the current LCOs culture oversimplify the interaction between extracellular matrix and cancer cells. Most LCO models solely consist of malignant cells, with fibroblasts and immune cells gradually disappearing during prolonged cultivation (126). Although growth factors and supplements can partially replicate the function of stromal cells, the lack of multicellular components in the tumor microenvironment remains a significant limitation of current LCO models. As such, they cannot be used to evaluate PD-1/PD-L1 immunotherapy or vascular-targeted drug treatments. With the advancement of co-culture, microfluidic and 3D printing technologies, numerous studies have been conducted on the interaction between tumor organoids and stromal cells to facilitate the identification of more targets and biomarkers for lung cancer treatment, as well as a superior drug evaluation platform. Despite many attempts by researchers to address this issue, enhancing mimicry in the tumor microenvironment remains a challenge.

2. LCOs serve as surrogate models for *in vitro* drug testing, enabling the prediction of patient-specific drug sensitivity and facilitating precision medicine. Due to variations in tumor tissue or cell sources, differences in culture media components across laboratories, variability in drug sensitivity detection methods for organoids, and diverse evaluation indicators of drug sensitivity (including IC_50_ based on cell activity detection reagents and area changes based on staining and imaging) (23, 35, 127), the complex operational steps, the organoid drug susceptibility testing results is suboptimal in reproducibility and accuracy. Organoid-based drug susceptibility testing requires more standardized culture and detection methods to gain greater clinical recognition. Liquid handling robots and automated high-throughput culture and analysis systems are among the new technologies that can optimize the utilization of tumor organoids (36, 128, 129). Furthermore, combining whole-exome sequencing, copy number assessment, and RNA sequencing can standardize the characterization of tumor organoids to ensure reproducibility and clinical efficacy consistency.

3. Organoids require integration with advanced biotechnologies to optimize their functionality (130). By combining organoids with single-cell technology, it is possible to determine whether tumor organoids can accurately represent the heterogeneity of lung cancer and gain insight into lung cancer development through organoid models (16, 45). Additionally, CRISPR/CAS9 gene-editing technology can be employed in conjunction with LCOs (79, 131, 132). This efficient system for editing organoids’ genes can be utilized to investigate the molecular mechanisms underlying lung cancer development, rapidly characterize genes related to cancer *in vivo*, replicate the entire process of tumor progression and metastasis, and explore its mechanism. Additionally, organoids can be combined with high-content imaging techniques to investigate drug mechanisms of action in a more sophisticated manner (133, 134). Organoids can be integrated with a genetic testing-based big data platform to identify biomarkers for drug efficacy and address the challenge of chemotherapy sensitivity that cannot be resolved by conventional genetic testing methods (77, 80). The incorporation of artificial intelligence analysis into organoid models enables accurate evaluation of drug effectiveness and facilitates new drug development (135, 136). The integration of organoid, microfluidic and 3D printing technologies enables the rapid establishment of a high-throughput organoid platform for drug screening and personalized medicine in cancer patients within one week (36, 128, 129, 137). Organoids hold immense potential for application in conjunction with state-of-the-art biomedical technologies, thereby enhancing their efficacy in research.

## 7 Conclusion

Organoid culture has already made a significant impact on the study of lung cancer. With its wider application, it has surpassed the limitations of previous clinical and laboratory studies and demonstrated extensive potential for use. By providing an easily manipulable model that allows for direct comparison of genotypes and phenotypes in a short period of time, organoid culture has opened up various experimental techniques that were previously unattainable. The utilization of LCOs enhances our fundamental comprehension of the initiation and progression, biology, and pathology of lung cancer, and is anticipated to be extensively applied in biomedical fields ranging from disease modeling to drug screening and personalized medicine. Nevertheless, despite its remarkable utility as a model system, the challenges confronting organoids cannot be disregarded. The resolution of these inquiries necessitates a multidisciplinary approach, requiring close collaboration between biologists, clinicians, and bioengineers to further investigate the numerous scientific questions surrounding LCOs. We firmly believe that organoids offer unique advantages in comprehending the onset, progression, and treatment of lung cancer. This will significantly advance both basic research and clinical treatment of cancer while greatly enhancing human health.

## Author contributions

YL: Writing – original draft, Writing – review & editing. YZ: Conceptualization, Methodology, Writing – review & editing. PC: Conceptualization, Formal Analysis, Resources, Writing – review & editing.
